# AI-based chest CT semantic segmentation algorithm enables semi-automated lung cancer surgery planning by recognizing anatomical variants of pulmonary vessels

**DOI:** 10.3389/fonc.2022.1021084

**Published:** 2022-10-13

**Authors:** Xiuyuan Chen, Hao Xu, Qingyi Qi, Chao Sun, Jian Jin, Heng Zhao, Xun Wang, Wenhan Weng, Shaodong Wang, Xizhao Sui, Zhenfan Wang, Chenyang Dai, Muyun Peng, Dawei Wang, Zenghao Hao, Yafen Huang, Xiang Wang, Liang Duan, Yuming Zhu, Nan Hong, Fan Yang

**Affiliations:** ^1^ Department of Thoracic Surgery, Peking University People’s Hospital, Beijing, China; ^2^ Thoracic Oncology Institute, Peking University People’s Hospital, Beijing, China; ^3^ Department of Radiology, Peking University People’s Hospital, Beijing, China; ^4^ Thoracic Surgery Department, Shanghai Pulmonary Hospital, Shanghai, China; ^5^ Thoracic Surgery Department, The Second Xiangya Hospital of Central South University, Changsha, China; ^6^ Institute of Advanced Research, Infervision Medical Technology Co., Ltd, Beijing, China

**Keywords:** pulmonary vessel, artificial intelligence, semantic segmentation, surgery planning, lung cancer

## Abstract

**Background:**

The recognition of anatomical variants is essential in preoperative planning for lung cancer surgery. Although three-dimensional (3-D) reconstruction provided an intuitive demonstration of the anatomical structure, the recognition process remains fully manual. To render a semiautomated approach for surgery planning, we developed an artificial intelligence (AI)–based chest CT semantic segmentation algorithm that recognizes pulmonary vessels on lobular or segmental levels. Hereby, we present a retrospective validation of the algorithm comparing surgeons’ performance.

**Methods:**

The semantic segmentation algorithm to be validated was trained on non-contrast CT scans from a single center. A retrospective pilot study was performed. An independent validation dataset was constituted by an arbitrary selection from patients who underwent lobectomy or segmentectomy in three institutions during Apr. 2020 to Jun. 2021. The golden standard of anatomical variants of each enrolled case was obtained via expert surgeons’ judgments based on chest CT, 3-D reconstruction, and surgical observation. The performance of the algorithm is compared against the performance of two junior thoracic surgery attendings based on chest CT.

**Results:**

A total of 27 cases were included in this study. The overall case-wise accuracy of the AI model was 82.8% in pulmonary vessels compared to 78.8% and 77.0% for the two surgeons, respectively. Segmental artery accuracy was 79.7%, 73.6%, and 72.7%; lobular vein accuracy was 96.3%, 96.3%, and 92.6% by the AI model and two surgeons, respectively. No statistical significance was found. In subgroup analysis, the anatomic structure-wise analysis of the AI algorithm showed a significant difference in accuracies between different lobes (p = 0.012). Higher AI accuracy in the right-upper lobe (RUL) and left-lower lobe (LLL) arteries was shown. A trend of better performance in non-contrast CT was also detected. Most recognition errors by the algorithm were the misclassification of LA^1+2^ and LA^3^. Radiological parameters did not exhibit a significant impact on the performance of both AI and surgeons.

**Conclusion:**

The semantic segmentation algorithm achieves the recognition of the segmental pulmonary artery and the lobular pulmonary vein. The performance of the model approximates that of junior thoracic surgery attendings. Our work provides a novel semiautomated surgery planning approach that is potentially beneficial to lung cancer patients.

## Introduction

Lung cancer is one of the leading causes of cancer-related morbidity and mortality worldwide, with an estimated 2.2 million new cases and 1.8 million deaths (1). With the increased frequency of computed tomography (CT) screening, especially thin-section CT, the early detection rate of small-sized lung cancer and ground-glass opacity has dramatically increased (2). Anatomic lobectomy and segmentectomy are the main curative treatments for early-stage lung cancer, especially segmentectomy, which preserves more lung tissue (3, 4). However, pulmonary arteries and veins are highly variable; understanding the anatomical structure of each patient during preoperative surgical planning is crucial yet challenging. The misclassification of segmental or even lobular vessels can occur even for experienced surgeons, which can lead to bleeding, increased surgical resection, or other catastrophic consequences.

Traditionally, chest CT is the most common tool for preoperative planning that typically consists of three steps: 1. 3-D reconstruction; 2. variant recognition; and 3. intraoperative projection. In the first step, surgeons rely on their own spatial imagination or 3-D reconstruction software to perform a 3-D reconstruction of anatomical structures. Second, normal anatomy and anatomical variations require careful identification, which relies heavily on the experience of the surgeon. Third, the surgeon needs to project the reconstruction of the preoperative 3-D anatomy to the intraoperative anatomy, that is, the surgeon matches and identifies the anatomy seen during the operation according to the preoperative 3-D reconstruction. All three steps rely solely on human effort, which impairs the accuracy and efficiency of preoperative planning. The recent development of artificial intelligence (AI), however, has shown potential in optimizing this practice.

AI algorithms have been widely applied in every aspect of medicine recently (5–9). From the screening of the pulmonary nodule (10) to the diagnosis of skin cancer (11) and diabetic retinopathy (12) and even in the development of new treatment drugs (13–15), utilizing the AI algorithm has been revolutionizing. Among all applications, the pattern recognition of medical imaging is the most reliable, of which semantic segmentation excels due to its interpretability and robustness in highly specialized tasks.

According to literatures, semantic segmentation algorithms are capable of detecting red blood cells for sickle cell disease in microscopic images (16); deciding the tumor border in pathological images (17, 18); recognizing the infection area of coronavirus disease of 2019 (COVID-19) lesions on chest CTs (19); distinguishing the brachia plexus, fetal head, and lymph node from ultrasound images (20); segmenting the thalamus, caudate nucleus, and lenticular nucleus in brain MRI (21); and diagnosing gastrointestinal cancer margins during endoscopy (22). Aiming to optimize the surgical planning process, we have previously developed a fully automated 3-D reconstruction algorithm (23) to classify and reconstruct the pulmonary artery and vein. The performance of the algorithm is the same as manual reconstruction. Although the granularity of these applications is coarse, the clinical significancy is solid.

In this study, we go one more step into the surgical planning process. We developed a fine-grained chest CT semantic segmentation algorithm that systematically identifies 18 segmental pulmonary arteries and 5 lobular pulmonary veins. We evaluated the independent performance of the algorithm using the CT data from 27 patients who had undergone lobectomy or segmentectomy at three medical institutions. This algorithm would facilitate the realization of a semiautomated surgical planning process, which is one of the backbones for the development of a fully automated thoracoscopic surgical system.

## Methods

### Patient enrollment

Patients who underwent lobectomy or segmentectomy at Peking University People’s Hospital, Shanghai Pulmonary Hospital, and the Second Xiangya Hospital of Central South University between Apr. 2020 to Jun. 2021 were retrospectively reviewed. The inclusion criteria were as follows: (1) preoperative thin-section (<1.25 mm), either non-enhanced chest CT images or contrast-enhanced CT chest CT images available, (2) the time interval between CT examination and surgery of less than 1 month, and (3) the video of surgery and preoperative 3-D reconstruction of pulmonary vessels and bronchi available. Among patients who met the above criteria, 27 cases representing most of the common lobectomies and segmentectomies were arbitrarily selected from three participating centers.

### Chest computed tomography acquisition

All enrolled patients underwent a chest CT examination at most a month prior to surgery. The whole lung scan from the thoracic entrance to the bottom of the lung was completed after one inhalation and holding breath using CT instruments from GE Healthcare (Chicago, IL, USA), Philips Healthcare (Amsterdam, Netherlands), Siemens Healthineers (Forchheim, Germany), and United Imaging (Shanghai, China). CT images were reconstructed by using different convolutional kernels with a layer thickness of less than 1.25 mm.

### Deep learning algorithm for automated semantic segmentation of pulmonary vessels

**Figure 1:**
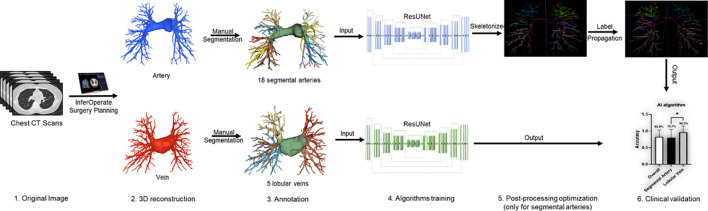
Workflow diagram of the deep learning (DL)-based pulmonary blood vessel segmentation system.

Based on the automatic 3-D reconstruction system of pulmonary blood vessels and bronchi (InferOperate Thoracic Surgery) (23), a pulmonary blood vessel semantic segmentation system was developed using deep learning (DL) algorithms for the automatic segmentation and name of the segmental pulmonary arteries and lobular pulmonary veins, aiming to provide guidance for surgery and promote the application of anatomical lobectomy and segmentectomy. In brief, segmental arteries and lobular veins were manually and concisely segmented based on the automatically constructed 3-D blood vessels by senior thoracic surgeons and then used for training the model. The ResUNet was utilized as the backbone, and the label propagation algorithm was employed to reduce the misclassification of segmental arteries and lobular veins; a schematic roadmap is shown in **Figure 1**. Label propagation is usually utilized to classify a massive number of unlabeled examples in the presence of a few labeled examples (24, 25). In our scenario, the label of each pixel is propagated to adjacent pixels according to the similarity. During each step of propagation, each pixel adds the label values propagated by its surrounding pixels according to the propagation probability and updates the probability distribution of their respective label types. By keeping the labels of known pixels unchanged at the initial value and then restarting a new round of propagation until each pixel of different categories is divided into a range, the color mixing of blood vessels could be well reduced. In this multicenter study, we validated the robustness and generalizability of the pulmonary blood vessel segmentation system by examining its segmentation performance on either plain CT scans or contrast-enhanced CT scans.

### The golden standard for anatomical structures compositions of targeted lesions

To quantitatively evaluate the performance of the pulmonary blood vessel segmentation system, the golden standard for calculating accuracy was firstly established by three senior thoracic surgeons with CT images, surgery videos, and preoperative 3-D reconstructions as references. Taking the clinical needs into account, the anatomical structures related to target lesions and their relationships were analyzed. Two senior thoracic surgeons reviewed these cases back to back, and disagreements would be settled by a third senior thoracic surgeon. To simplify the establishment procedure, possible anatomical variants that represent the spatial pattern of relevant anatomical structures were enumerated in a table, which is inherited from our previous study with minor simplification (23) and made available to the surgeons (**Supplementary Table 1**).

### Reader study

To comprehensively analyze the performance of the pulmonary blood vessel segmentation system, we invited two junior attendings in thoracic surgery at Peking University People’s Hospital to participate in a reader study in comparison to the AI algorithm. The recognition by both the algorithm and junior attendings is based on chest CT. The recognized anatomical variant is selected or described based on **Supplementary Table 1**. By comparing with the golden standard, recognition accuracy was calculated to evaluate their performance and compare it with the DL-based system.

### Evaluation index and statistical analysis

Accuracy was utilized as the evaluation index in the independent performance test and reader study, including anatomical structure-wise accuracy and case-wise accuracy. The former was defined as the correctly segmented/recognized targeted pulmonary vessel structures in the 3-D reconstruction divided by the total related structures, while the latter was achieved by averaging the anatomical structure-wise accuracy of each case. Patients’ demographics and clinical features (age, sex, and smoking history), tumor characteristics (tumor location, tumor size, and histology), and surgery characteristics (blood loss and operation time) were analyzed. Continuous data with normality distribution and the homogeneity of variance were analyzed using one-way ANOVA; otherwise, the Mann–Whitney test or Kruskal–Wallis test was utilized. Meanwhile, the categorical variables were processed by chi-square or Fisher’s exact tests as appropriate.

## Results

### Clinical characteristics

To validate the performance and practice of the DL-based semantic segmentation algorithm, we arbitrarily selected 27 patients who underwent lobectomy or segmentectomy from Apr. 2020 to Jun. 2021 in three medical centers, among which nine were enrolled from Peking University People’s Hospital, nine were enrolled from Shanghai Pulmonary Hospital, and nine were enrolled from The Second Xiangya Hospital of Central South University. The clinical characteristics are delineated in **Table 1**. The median age was 59 with the interquartile range (IQR) from 53.5 to 64.5 years. A total of 14 patients (51.85%) were women, and 13 (48.15%) were men. Most of the enrolled patients were non-smokers (88.89%). Five of the enrolled cases were pathologically diagnosed with benign lesions and 22 with malignant ones (MIA or invasive adenocarcinoma). Regarding the location of lesions, 10 RUL, 4 right-middle lobe (RML), 5 right-lower lobe (RLL), 6 left-upper lobe (LUL), and 2 LLL were included. With respect to surgical procedures, 10 received segmentectomy and 17 underwent lobectomy. No significant differences between baseline data were found across the three institutions except for blood loss (30 ml vs. 50 ml vs. 50 ml, p = 0.006). All enrolled cases were successfully processed by the semantic segmentation algorithm without systemic failures. The outputs of the algorithm are demonstrated in **Supplementary Figures 1–6**. The median inference time was 100 s.

**Table 1:** Clinical characteristics of the validation dataset.

Variable	Total	Center A	Center B	Center C	p-value
Number of cases, n	27	9	9	9	
Age, median (IQR), years	59 [40, 75]	62 (49–63)	58 (55–63)	57 (53–67)	0.981
Sex, n(%)					0.145
Female	14 (51.9)	3 (33.3)	4 (44.4)	7 (77.8)	
Male	13 (48.1)	6 (66.7)	5 (55.6)	2 (22.2)	
Smoking history, n(%)					
Yes	3 (11.1)	1 (11.1)	1 (11.1)	1 (11.1)	1
No	24 (88.9)	8	8	8	
Histology, n(%)					0.267
Benign lesion	5 (18.5)	0 (0)	2 (22.2)	3 (33.3)	
MIA*	7 (25.9)	4 (44.4)	1 (11.1)	2 (22.2)	
Invasive adenocarcinoma	15 (55.6)	5 (55.6)	6 (66.7)	4 (44.4)	
Tumor location^**^					0.569
RUL	10 (37.0)	4 (44.4)	1 (11.1)	5 (55.6)	
RML	4 (14.8)	1 (11.1)	2 (22.2)	1 (11.1)	
RLL	5 (18.5)	1 (11.1)	3 (33.3)	1 (11.1)	
LUL	6 (22.2)	2 (22.2)	3 (33.3)	1 (11.1)	
LLL	2 (7.4)	1 (11.1)	0 (0)	1 (11.1)	
Surgery type, n(%)					
Segmentectomy	10 (37)				
Lobectomy	17 (63)				
Blood loss, median (IQR), ml	50 [20, 50]	30 [20, 50]	50 [40, 50]	50 [50, 50]	0.006
Operation time, median (IQR), min	110 [60, 195]	120 [110, 146]	100 [90, 120]	110 [100, 120]	0.355
CT instrument manufacturer					<0.05
GE	6 (22.2)	1 (11.1)	5 (55.6)	0 (0)	
Siemens	15 (55.6)	6 (66.7)	0 (0)	9 (100)	
Philips	4 (14.8)	0 (0)	4 (44.4)	0 (0)	
UIH	2 (7.4)	2 (22.2)	0 (0)	0 (0)	
Chest CT slice thickness, mm					0.072
0.625	3 (11.1)	0 (0)	3 (33.3)	0 (0)	
0.8	1 (3.7)	0 (0)	0 (0)	1 (11.1)	
1	20 (74.1)	8 (88.9)	4 (44.4)	8 (88.9)	
1.25	3 (11.1)	1 (11.1)	2 (22.2)	0 (0)	
Imaging convolutional kernels					<0.05
stnd	3 (11.1)	1 (11.1)	2 (22.2)	0 (0)	
Lung	3 (11.1)	0 (0)	3 (33.3)	0 (0)	
B70f	7 (25.9)	0 (0)	0 (0)	7 (77.8)	
Br40d\3	5 (18.5)	5 (55.6)	0 (0)	0 (0)	
Br60f\3	1 (3.7)	0 (0)	0 (0)	1 (11.1)	
BI64d\3	1 (3.7)	1 (11.1)	0 (0)	0 (0)	
BI57d\2	1 (3.7)	0 (0)	0 (0)	1 (11.1)	
Lung iDose(3)	1 (3.7)	0 (0)	1 (11.1)	0 (0)	
Lung iDose(4)	3 (11.1)	0 (0)	3 (33.3)	0 (0)	
Soft B	2 (7.4)	2 (22.2)	0 (0)	0 (0)	

* Microinvasive adenocarcinoma.

** RUL, right-upper lobe; RML, right-middle lobe; RLL, right-lower lobe; LUL, left- upper lobe; LLL, left-lower lobe.

### Performance of the semiautomated surgical planning algorithm

**Figure 2:**
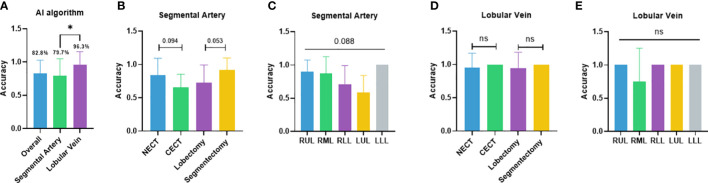
Performance of the semiautomated surgical planning algorithm. **(A)** Case-wise overall accuracy and accuracy in arteries and veins; **(B)** artery accuracy comparison between contrast and non-contrast CT, lobectomy and segmentectomy; **(C)** artery accuracy comparison between different lobes; **(D)** vein accuracy comparison between contrast and non-contrast CT, lobectomy, and segmentectomy; **(E)** vein accuracy comparison between different lobes. NECT, non-enhanced CT; CECT, contrast-enhanced CT; RUL, right-upper lobe; RML, right-middle lobe; RLL, right-lower lobe; LUL, left-upper lobe; LLL, left- lower lobe. *p<0.05; ns non-significant.

The independent performance of the DL-based semantic segmentation algorithm in classifying segmental arteries and lobular veins was first validated. The overall case-wise accuracy of the algorithm was 82.8% (**Figure 2A**). In the segmental artery recognition task, the accuracy was 79.7% (**Figure 2A**). Higher accuracy was seen in non-contrast CT (83.8% vs. 65.3%, p = 0.094) and segmentectomies (91.7% vs. 72.7%, p = 0.053) when compared to contrast CT scans and lobectomies, respectively (**Figure 2B**). Arteries in the LLL showed the highest recognition accuracy at 100.0%, followed by the RUL (90.0%), the RML (87.5%), the RLL (70.5%), and the LUL (58.3%) (**Figure 2C**). In the lobular vein recognition task, the accuracy was 96.3% (**Figure 2A**). In subgroup analyses, the accuracy reached 95.2% and 100.0% on non-contrast CT and contrast CT scans, respectively; similar accuracies (94.1% vs. 100.0%) were observed for lobectomy and segmentectomy cases (**Figure 2D**). Of note, the RUL, RLL, LUL, and LLL reached 100.0% accuracy while in the RML, the accuracy was lower (75.0%) (**Figure 2E**).

### Performance of junior thoracic surgery attendings

**Figure 3:**
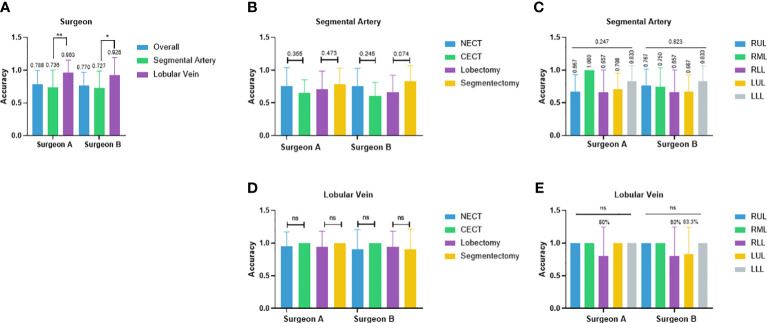
Performance of two junior thoracic surgery attendings. **(A)** Case-wise overall accuracy and accuracy in arteries and veins; **(B)** Artery accuracy comparison between contrast and non-contrast CT, lobectomy, and segmentectomy; **(C)** artery accuracy comparison between different lobes; **(D)** vein accuracy comparison between contrast and non-contrast CT, lobectomy, and segmentectomy; **(E)** vein accuracy comparison between different lobes. NECT, non-enhanced CT; CECT, contrast-enhanced CT; RUL, right-upper lobe; RML, right-middle lobe; RLL, right-lower lobe; LUL, left-upper lobe; LLL, left-lower lobe. *p<0.05, **p<0.01, ns non-significant.

Two junior attendings participated in the reader study for recognizing pulmonary vascular anatomical structures and obtained the overall accuracy of 78.8% and 77.0%, respectively (**Figure 3A**). In the segmental artery recognition task, the accuracy was 73.6% and 72.7%, respectively. Accuracies slightly favored non-contrast over contrast CT (76.0% vs. 65.3%, p = 0.355; 76.0% vs. 61.1%, p = 0.245) and favored segmentectomy over lobectomy (78.3% vs. 70.8%, p = 0.473; 83.3% vs. 66.4%, p = 0.074) (**Figure 3B**). No significant difference across lobes were shown (RUL 66.7%, RML 100.0%, RLL 65.7%, LUL 70.8%, LLL 83.3%, p = 0.247; RUL 76.7%, RML 75.0%, RLL 65.7%, LUL 66.7%, LLL 83.3%, p = 0.823) (**Figure 3C**). In the lobular vein recognition task, the accuracy was 96.3% and 92.6%, respectively (**Figure 3A**). No significant difference between non-contrast and contrast CT was shown (**Figure 3D**). The accuracy of the RLL was 80% in both surgeons and LUL 83.3% in surgeon B (**Figure 3E**). All other lobular vein recognitions reached 100.0% accuracy (**Figure 3E**).

### Performance comparison between the artificial intelligence algorithm and surgeons’ performance

**Figure 4:**
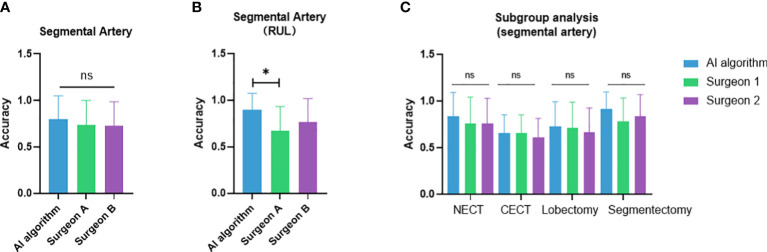
Performance comparison between the artificial intelligence algorithm and two surgeons. **(A)** Three-way analysis between three groups; **(B)** Pairwise analysis showed significantly lower accuracy in the RUL of surgeon A compared to the AI algorithm; **(C)** subgroup analysis between non-contrast vs. contrast CT and lobectomy vs. segmentectomy. NECT, non-enhanced CT; CECT, contrast-enhanced CT. *p<0.05; ns, non-significant.

**Figure 5:**
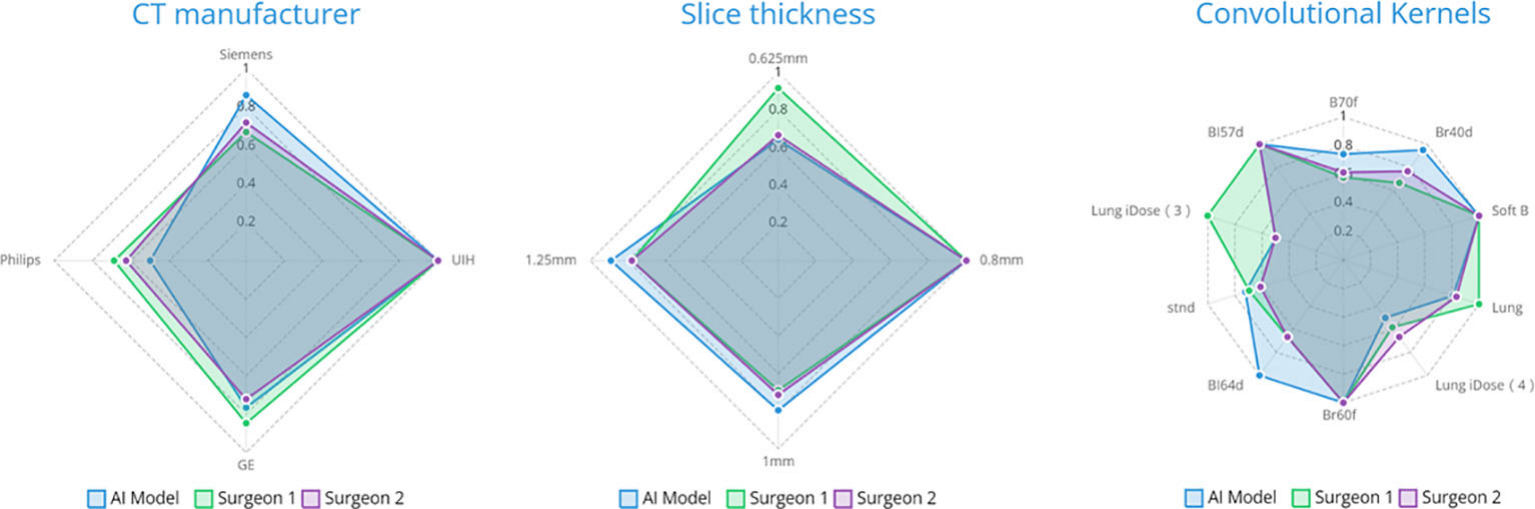
The impact of radiological parameters on accuracy on three parties.

The case-wise artery recognition accuracy showed no significant difference between the AI algorithm and two surgeons in three-way analysis (**Figure 4A**). Upon further pairwise analysis, surgeon A showed significantly lower accuracy in the RUL (p = 0.037) (**Figure 4B**). In non-contrast CT, contrast CT, lobectomy and segmentectomy subgroups, no significant differences were shown across the AI algorithm and two surgeons (p = 0.493, 0.825, 0.872, 0.396) (**Figure 4C**). Different CT manufacturers, slice thicknesses, or convolutional kernels showed no significant impact on the AI algorithm and two surgeons (**Figure 5**).

### Error analysis of artificial intelligence and human recognition

Error analyses were performed by regrouping the results at the segmental structure level instead of the case level. All recognition errors by the algorithm are listed in **Supplementary Figures 7–18**. Most errors were observed in segmental arteries, while few errors were seen in lobular veins. The analysis of the AI recognition showed significant differences across different segmental arteries (p = 0.002) (**Table 2**), among which RA^1^, RA^2^, RA^3^, RA^4+5^, RA^6^, LA^6^, and LA^8-10^ showed higher accuracies. Failure in distinguishing LA^1+2^ and LA^3^ was the most frequently seen error in the algorithm; accuracy in right-lower basal segmental arteries was also below 50%. The error of two surgeons was analyzed on a structure-wise basis (**Table 2**). For surgeon A, a significant difference across different segmental arteries was detected (p = 0.033), among which RA^1^ and RA^4+5^ showed higher accuracy and LA^1+2^ showed significant lower accuracy. The accuracies of RA^2^, RA^6^, LA^4^, LA^5^, and LA^6^ were also below 50%. For surgeon B, no significant differences across different segmental arteries were detected (p = 0.083). The accuracies of LA^1+2^, LA^4^, LA^5^, and LA^6^ were less than 50%.

**Table 2:** Accuracy of the segmental artery in structure-wise error analysis.

Tumor location*	Segmental artery	AI	Surgeon A	Surgeon B
RUL	A1	1.00	0.86	1.00
	A2	0.80	0.30	0.50
	A3	0.75	0.50	0.50
RML	A4, A5	0.86	1.00	0.71
RLL	A6	0.75	0.33	1.00
	A7–10	0.20	0.60	0.60
LUL	A1+2	0.00	0.25	0.25
	A3	0.40	0.80	0.80
	A4, A5	0.60	0.40	0.20
LLL	A6	1.00	0.00	0.00
	A8–10	1.00	1.00	1.00
In group p-value		0.002	0.033	0.083

* RUL, right-upper lobe; RML, right-middle lobe; RLL, right-lower lobe; LUL, left-upper lobe; LLL, left-lower lobe.

## Discussion

In this study, we first reported a CT-based pulmonary vessel semantic segmentation algorithm for semiautomated surgical planning and comprehensively evaluated its clinical value by performing a validation study on multicenter datasets. Our results showed that the independent performance of the semantic segmentation algorithm approximated that of junior attendings in thoracic surgery. The overall recognition accuracy ranged from 70% to 80%. Subgroup analyses uncovered the possible influential factors on algorithm performance, including the usage of a contrast agent, radiation doses, the CT instrument manufacturer, and reconstruction convolutional kernels, and explored its potential applicable scenarios, such as lobectomy and segmentectomy.

A comprehensive understanding of the blood vessel structure, especially the artery structure, is the most crucial task in preoperative planning to minimize the chance of intraoperative bleeding and misresection (26–28). Given the fact that arteries are more hazardous than veins in surgeries and require delicate recognition for precise resection, in addition to the enormous number of vein variants on a segmental scale that cannot be fully represented due to the limited amount of training data, we thus split the surgical planning into two separate recognition tasks: segmental artery recognition and lobular vein recognition by developing a model that realizes the fine-grained recognition on arteries and relatively coarse-grained recognition on veins. Model performance was also evaluated by segmental artery recognition accuracy and lobular vein recognition accuracy on both case-wise and structure-wise.

In artery recognition, the algorithm showed similar accuracy compared to surgeons. It is worth notice that two parties exhibited complementary superiorities across lobes. The algorithm excels in the RUL, while surgeons excel in bilateral basal segments. Our result indicates a valid clinical application of this version of algorithm in assisting surgical planning in certain lobes, especially for junior thoracic surgeons. Vein recognition showed high accuracy in both parties; the algorithm may help in verifying the surgeon’s recognition. Corroborating our result, our previous study^9^ showed that the accuracy of the manual identification of the anatomical variant by thoracic surgeons using automated 3-D reconstruction is 85%, which is similar to our semiautomated approach. As for the time efficiency, compared to our previous study^9^ that showed a median recognition time of 120 s by surgeons using 3-D reconstruction images, the human recognition of anatomical structure in this research is no longer needed, and the variant recognition time can be ignored, which is accomplished in less than 5 s.

When analyzing the possible influential factors for this semantic segmentation algorithm, we found the similar trend of lower accuracy in the contrast CT subgroup for both the AI algorithm and human performance. Contrast CTs are usually used in cases in which blood vessels were overlayed by the lesion. Such situation is more challenging for the algorithm than for surgeons. Since the model was also trained based on mostly non-contrast CT, the extra information provided by contrast CT may have not been fully utilized.

In terms of applicable scenarios, higher recognition accuracy was observed in segmentectomy compared with lobectomy for both the AI algorithm and the thoracic surgeon. Meanwhile, the error analysis indicated that the algorithm struggled in certain segmental arteries (LA^1+2^, right basal segmental arteries, etc.) and surgeons struggled more in bilateral upper lobe arteries (RA^2^, RA^3^, and LA^1+2^). Our preliminary results indicated a better performance of the AI algorithm on segmentectomy. Considering the possible bias introduced by a limited number of cases and unevenly distributed surgery types, a larger dataset needs to be employed to further confirm the observation. As for the inferior performance on LA^1+2^ and right basal segmental arteries, more similar training data are needed to enhance the model performance.

A different performance between the AI algorithm and surgeons was observed across lobes that may reflect their difference in mechanics. The algorithm performed well in RUL, while surgeons did not. On one hand, the RUL has relatively well-defined anatomical patterns that are easier for the algorithm to learn, while the 3-D structure is difficult for humans to imagine. On the other hand, the RLL, especially basal segmental arteries, have more anatomical variants that cannot be completely defined and represented during model training, while the imagination of a 3-D structure is relatively easy for humans; thus, surgeons perform better than the algorithm.

To our knowledge, this is the first study that validated the performance of a semiautomated surgical planning algorithm based on the semantic segmentation of CT imaging. Recently, a number of attempts of using semantic segmentation in medical imaging have been made. We have seen its application from the microscopic scale of segmenting red blood cells for sickle cell disease (16) to segmenting the COVID-19 infection area from chest CT (19) or lesions from endoscope images (22). However, the above-mentioned applications are mostly single-labeled tasks, using a large training set to achieve an organ or lesion recognition task. Our work is the first in the class semantic segmentation application that systemically presented the blood vessel anatomy of an organ. The complexity of this task is yet unparalleled.

In conclusion, the semiautomated surgical planning algorithm achieves similar accuracy in both segmental artery and lobular vein recognition compared to the junior attendings of thoracic surgery. As a continuation of our previous fully automated 3-D reconstruction study, the addition of a fine-grained semantic segmentation algorithm greatly enhanced their competency and practicality in aiding accurate preoperative planning and even made intraoperative intelligent interaction possible. With increased training data and refined labeling in the near future, the model will achieve higher accuracy and benefit both surgeons and patients in lobectomy and segmentectomy. Since the study is based on a small sample size, the promising results are to be confirmed in a large-scale validation study in the future.

## Data availability statement

The raw data supporting the conclusions of this article will be made available by the authors, without undue reservation.

## Ethics statement

The studies involving human participants were reviewed and approved by Peking University People’s Hospital ethics committee (2022PHB011-002). Written informed consent for participation was not required for this study in accordance with the national legislation and the institutional requirements.

## Author contributions

XC, HX, and DW: drafting manuscript, data labeling, result analyzing, model training; QQ, CS, CD, and MP: dicom data collection, data labeling; JJ, HZ, and ZW: data labeling; SW: label consultation; XuW and WW: reader study; XS, XiW, and LD: golden standard; ZH and YH: model training, label validation; YZ, NH, and FY: draft revision. All authors contributed to the article and approved the submitted version.

## Funding

The work is funded by the Ministry of Science and Technology of the People’s Republic of China, Grant/Award Number: 2020AAA0109600. This research is also supported by grants from Shanghai Hospital Development Center (SHDC12021111).

## Acknowledgments

We would like to thank Tong Zou for his contribution to this work.

## Conflict of interest

Authors DW, ZH, and YH were employed by Infervision Medical Technology Co., Ltd.

The remaining authors declare that the research was conducted in the absence of any commercial or financial relationships that could be construed as a potential conflict of interest.

The reviewer YH declared a shared parent affiliation with the author(s) XC, HX, QQ, CS, JJ, HZ, XuW, WW, SW, XS, ZW, NH and FY to the handling editor at the time of review.

## Publisher’s note

All claims expressed in this article are solely those of the authors and do not necessarily represent those of their affiliated organizations, or those of the publisher, the editors and the reviewers. Any product that may be evaluated in this article, or claim that may be made by its manufacturer, is not guaranteed or endorsed by the publisher.
