# Supplementary material for: AI-based chest CT semantic segmentation algorithm enables semi-automated lung cancer surgery planning by recognizing anatomical variants of pulmonary vessels
Source: Front Oncol. 2022 Oct 13;12:1021084. doi: 10.3389/fonc.2022.1021084 (PMC9621115; doi:10.3389/fonc.2022.1021084)
Supplement: Supplementary file 1 [file Presentation_1.pptx]

## Slide 1
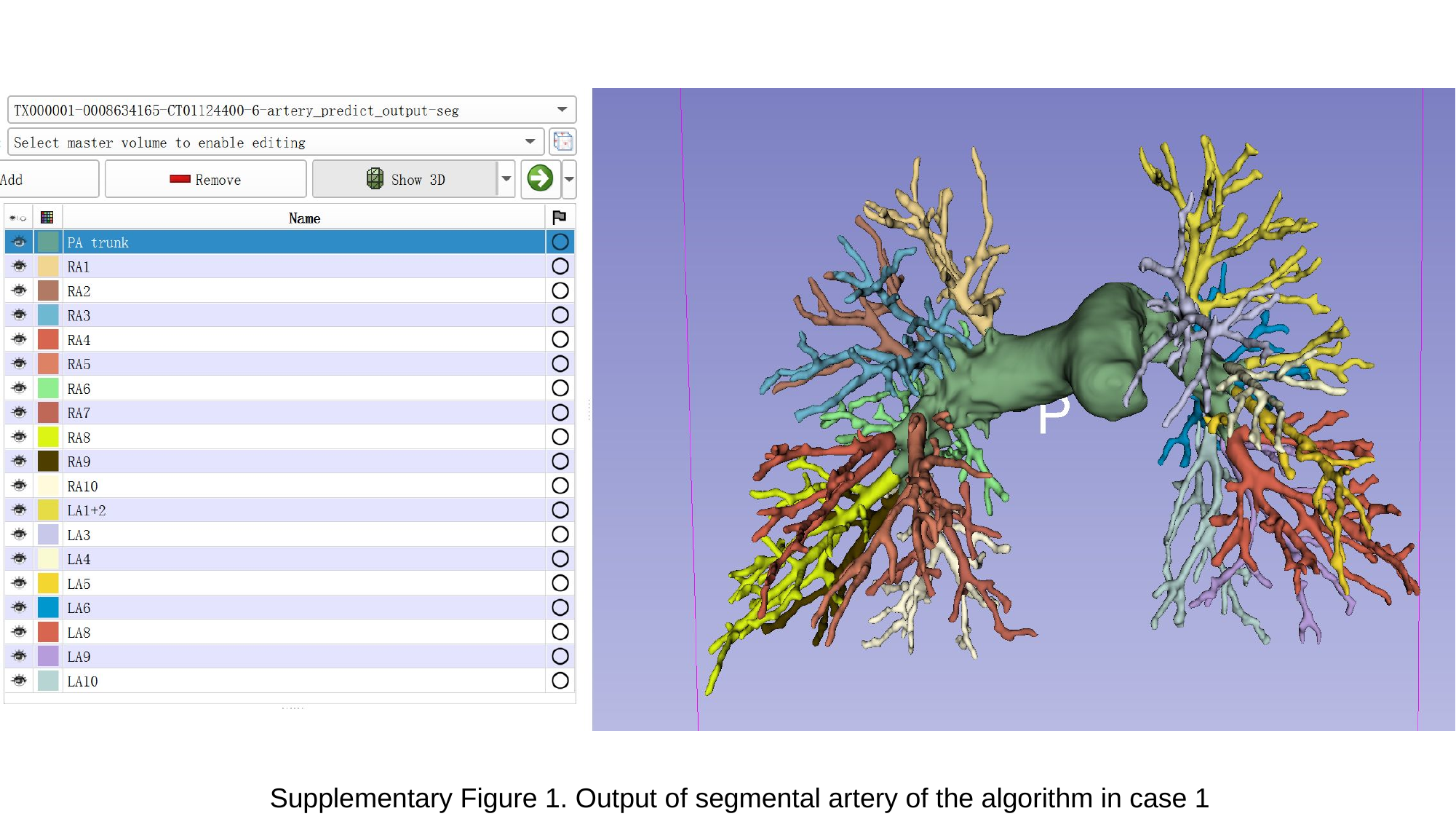

Supplementary Figure 1. Output of segmental artery of the algorithm in case 1

## Slide 2
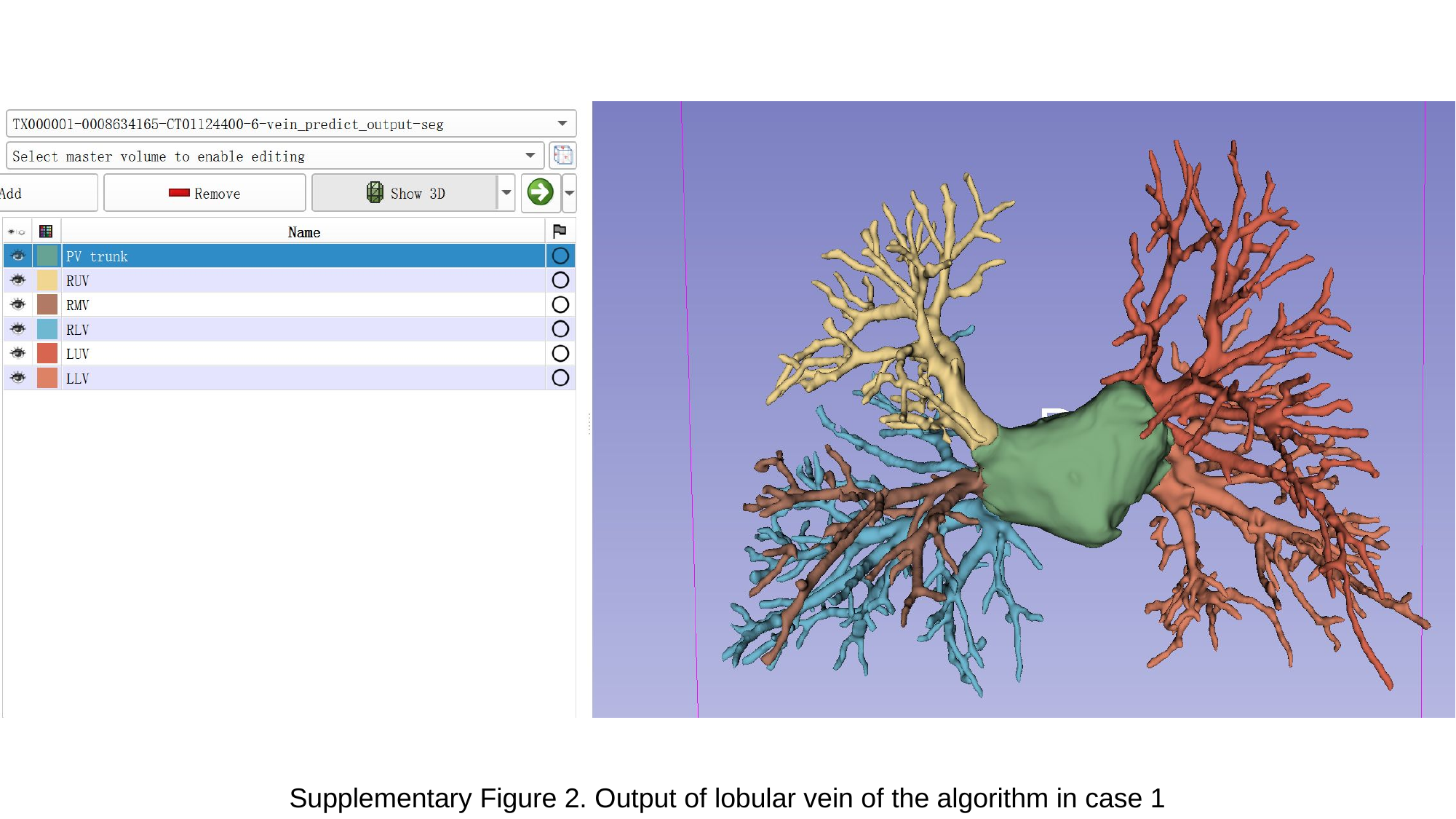

Supplementary Figure 2. Output of lobular vein of the algorithm in case 1

## Slide 3
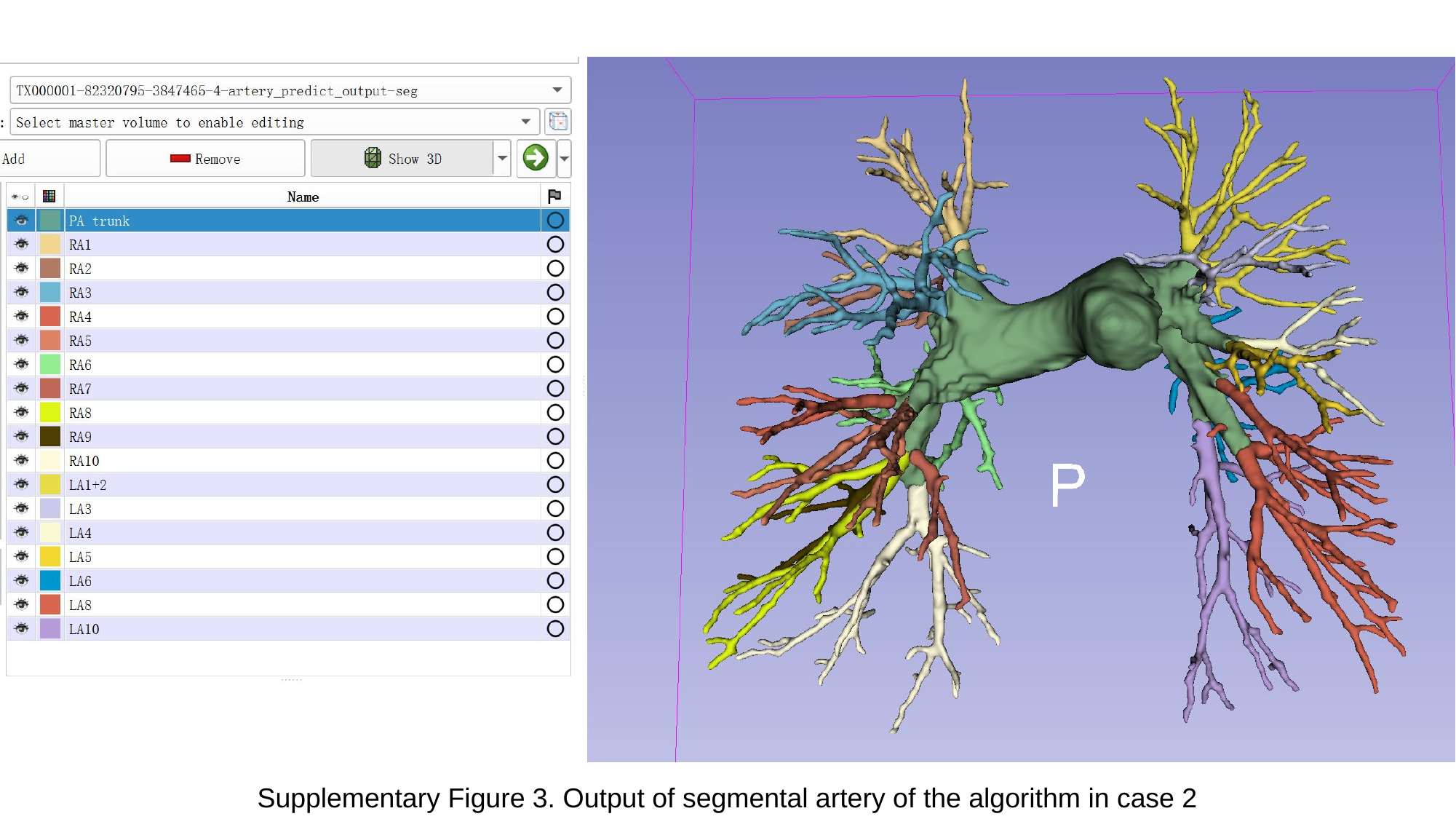

Supplementary Figure 3. Output of segmental artery of the algorithm in case 2

## Slide 4
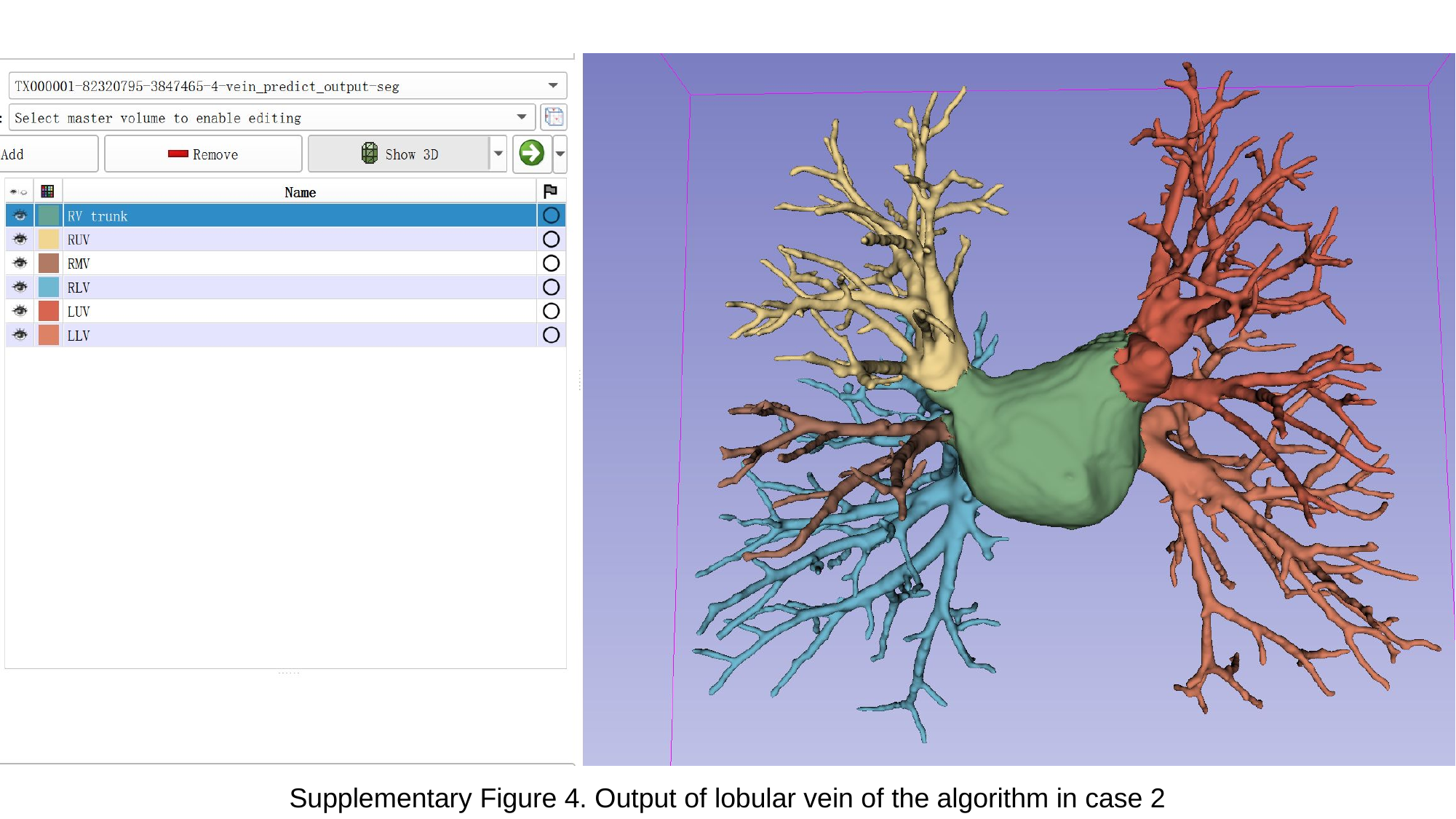

Supplementary Figure 4. Output of lobular vein of the algorithm in case 2

## Slide 5
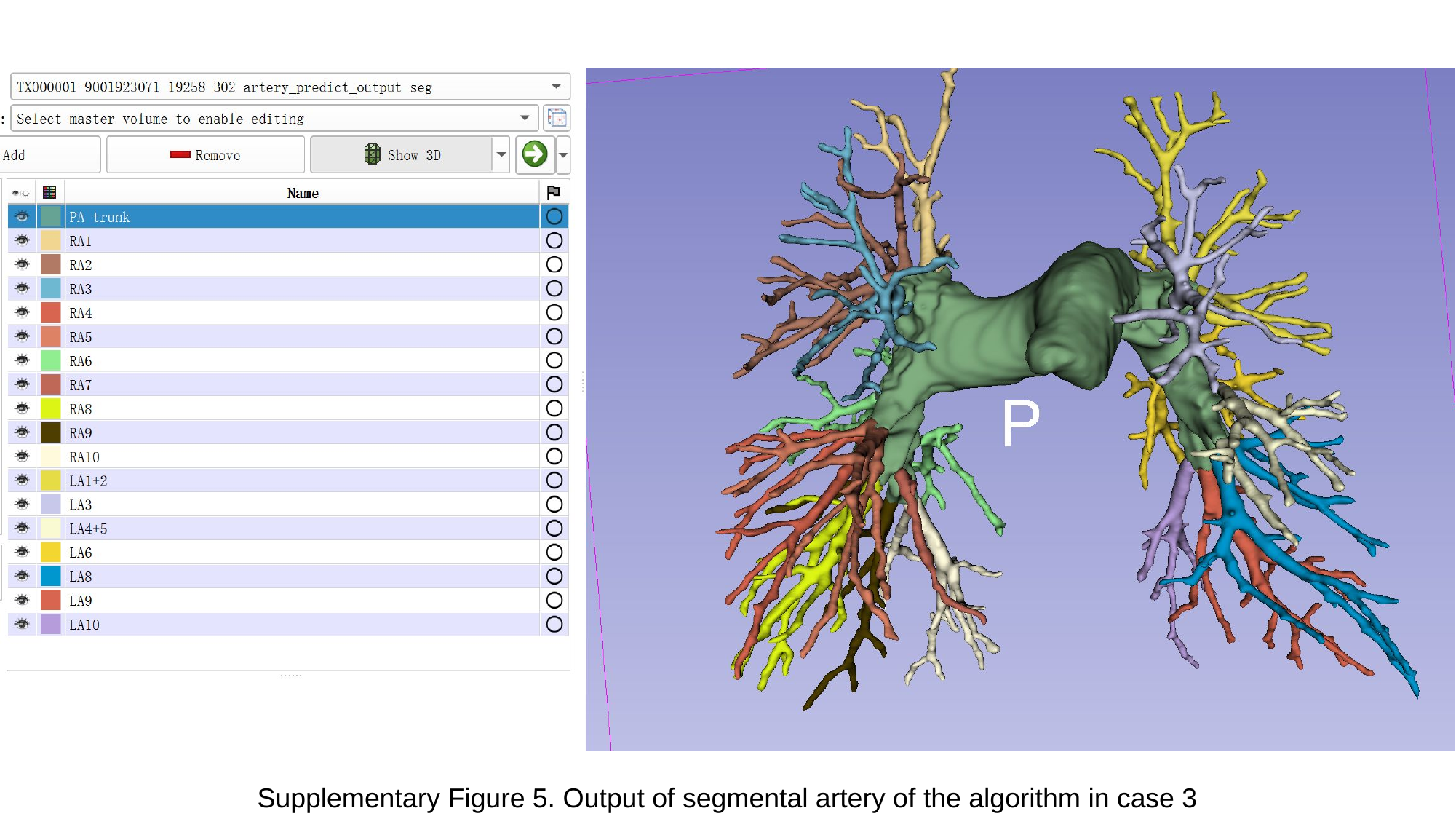

Supplementary Figure 5. Output of segmental artery of the algorithm in case 3

## Slide 6
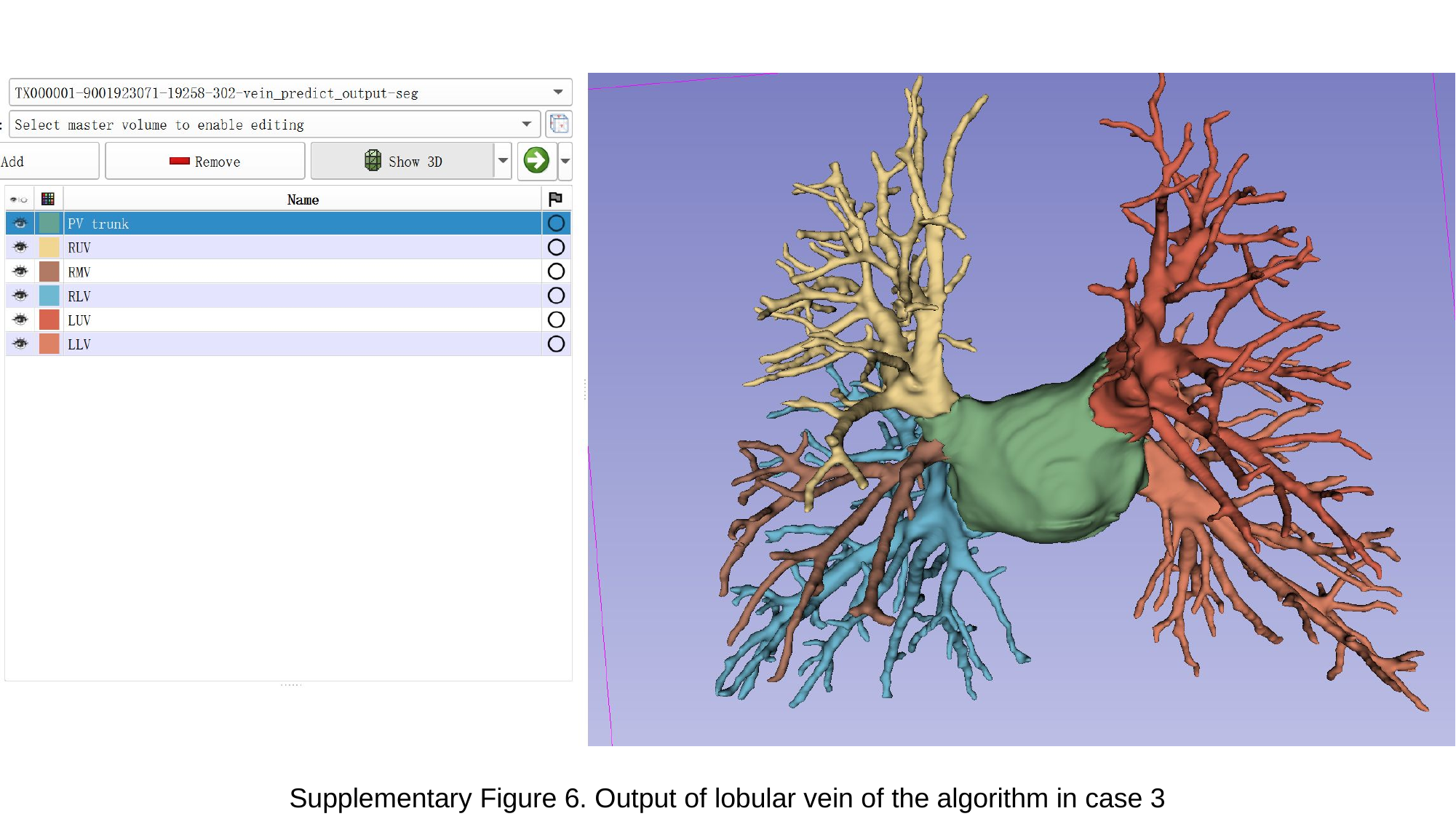

Supplementary Figure 6. Output of lobular vein of the algorithm in case 3

## Slide 7
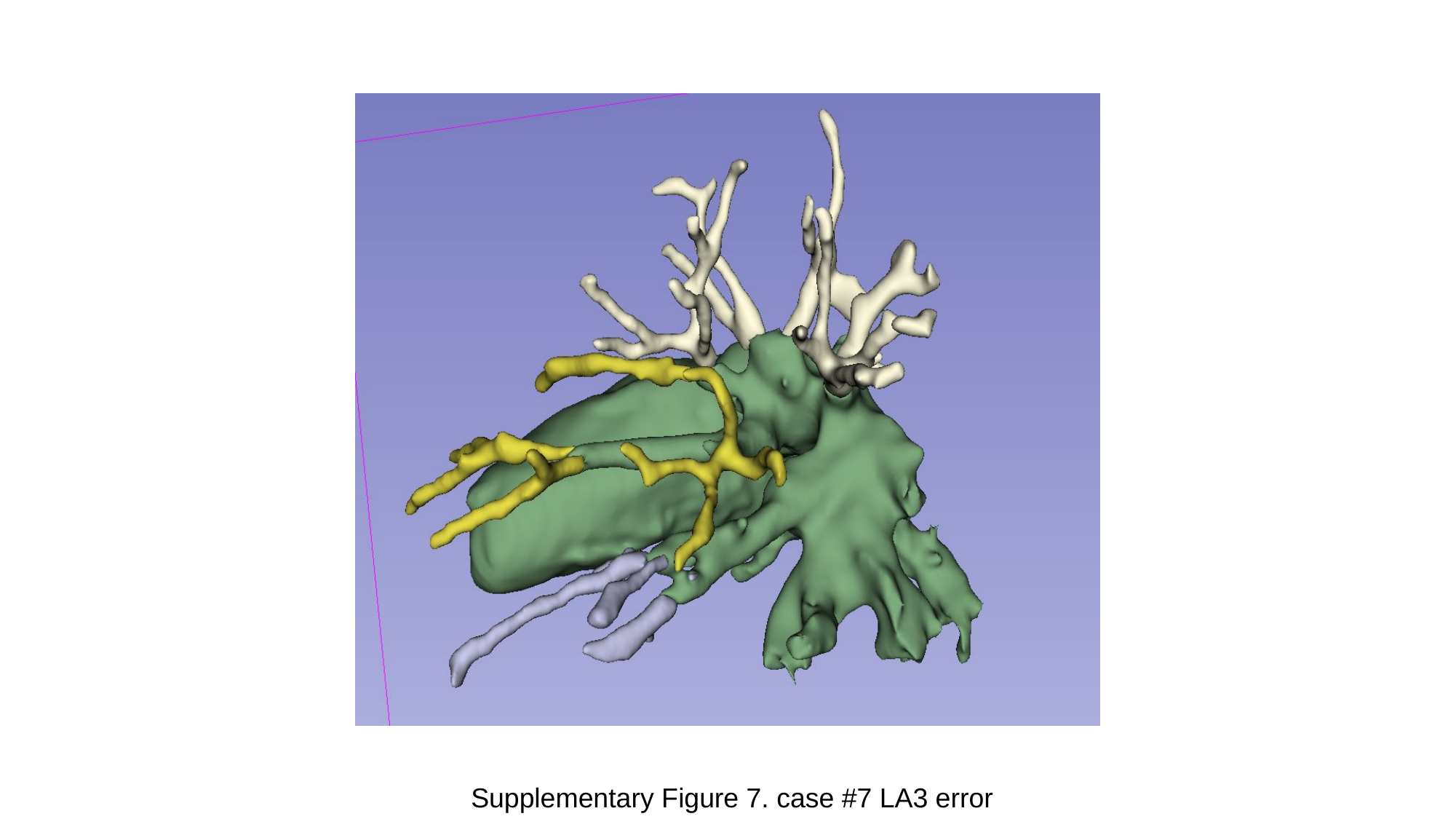

Supplementary Figure 7. case #7 LA3 error

## Slide 8
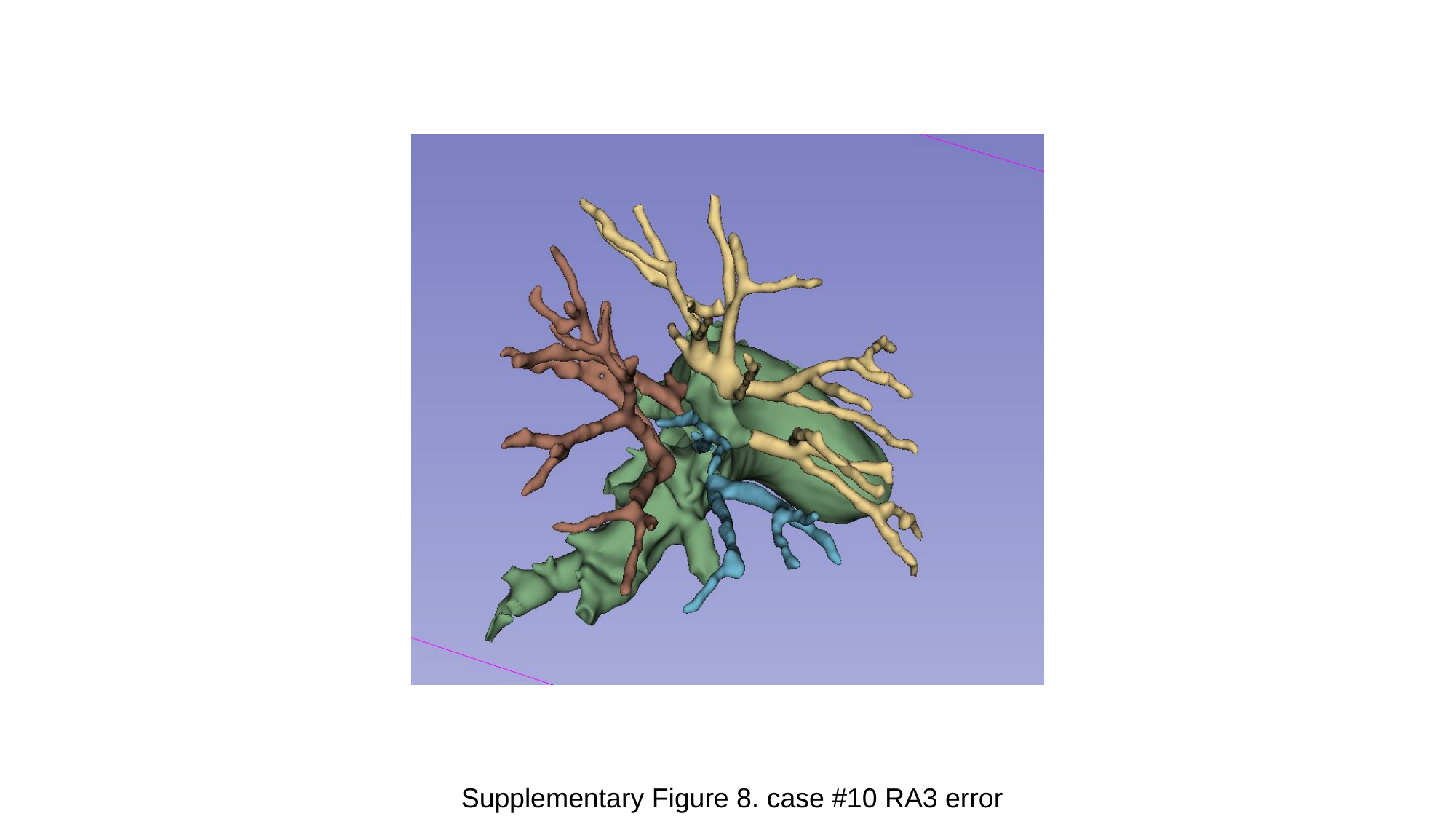

Supplementary Figure 8. case #10 RA3 error

## Slide 9
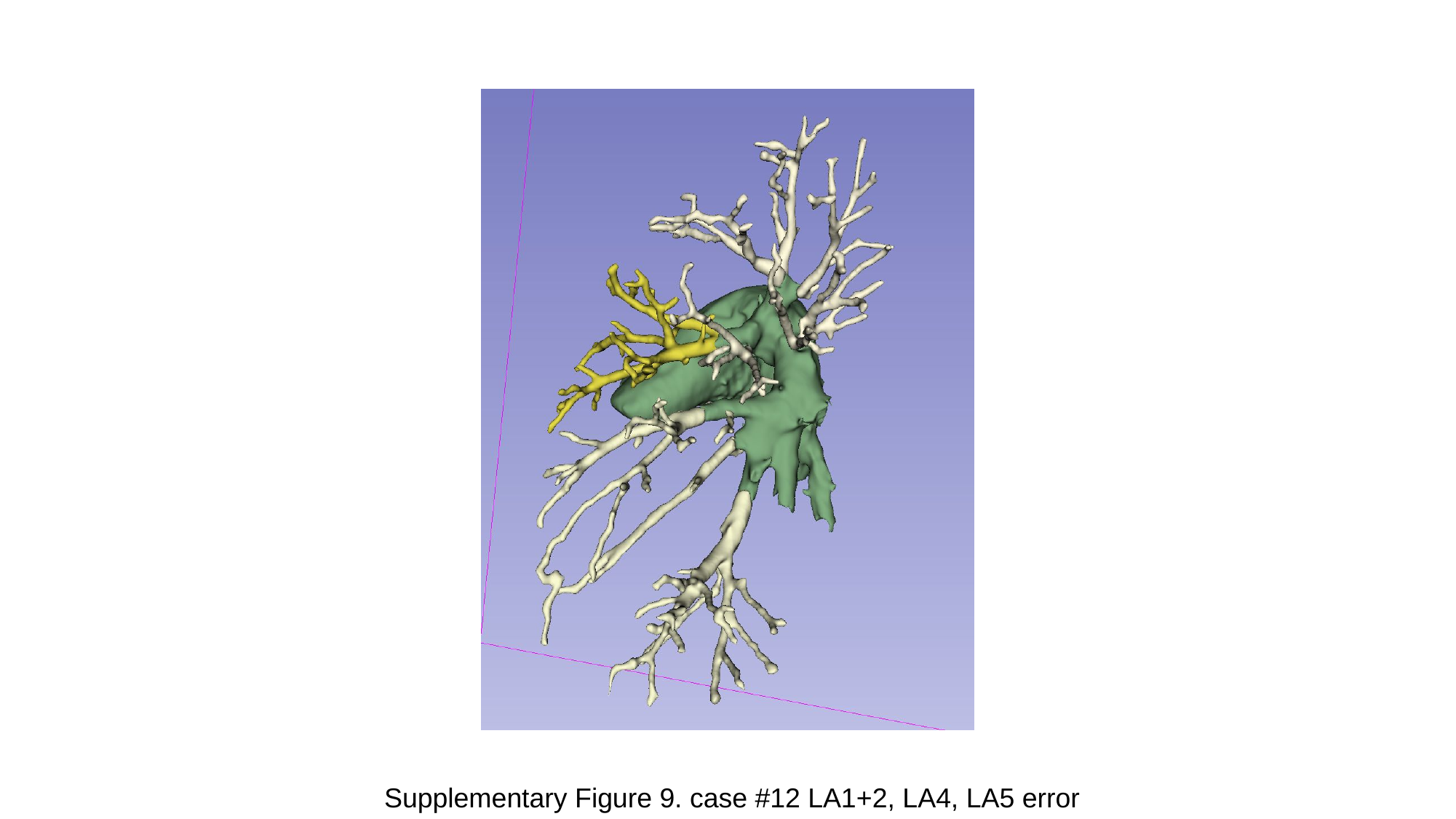

Supplementary Figure 9. case #12 LA1+2, LA4, LA5 error

## Slide 10
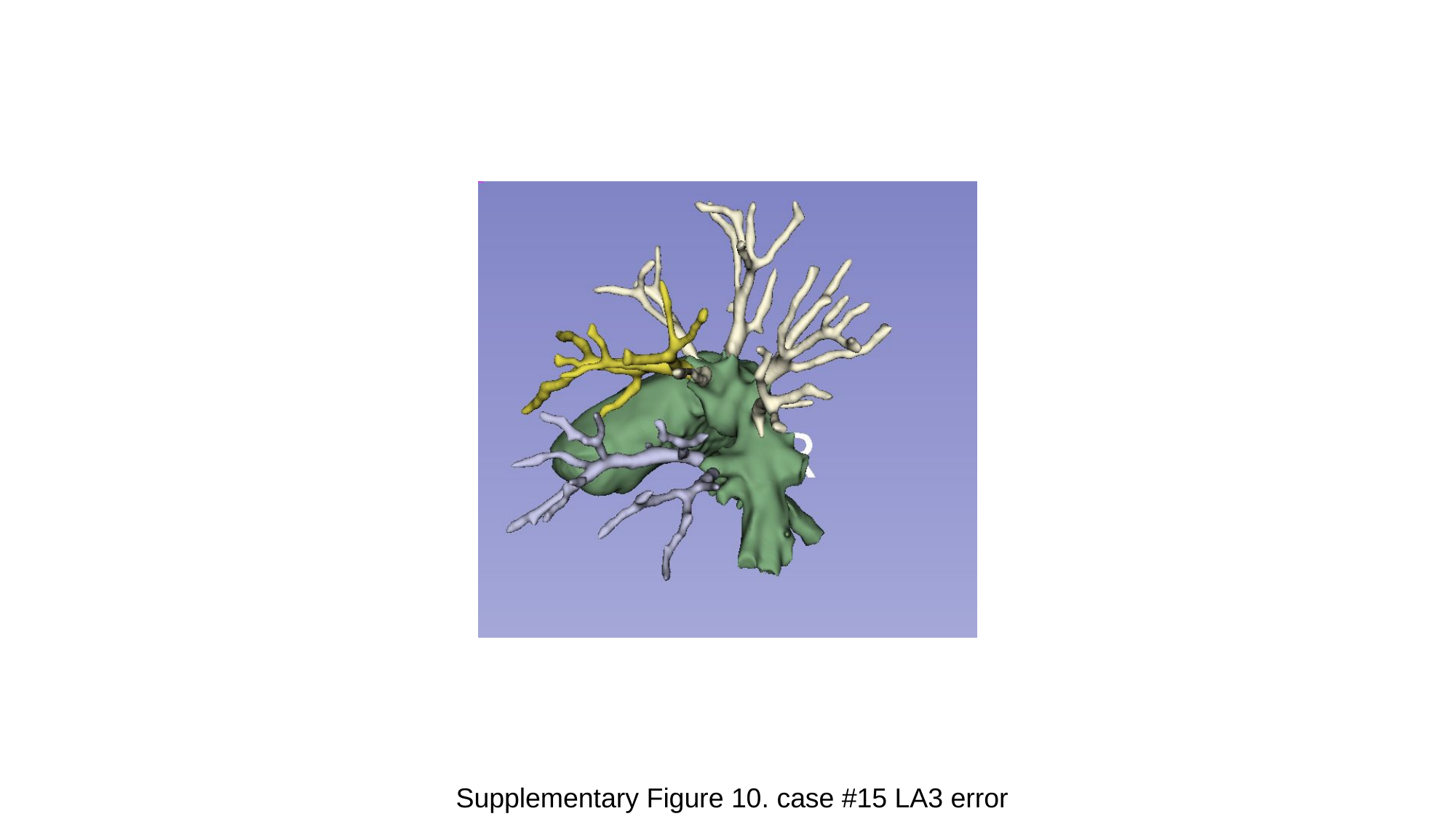

Supplementary Figure 10. case #15 LA3 error

## Slide 11
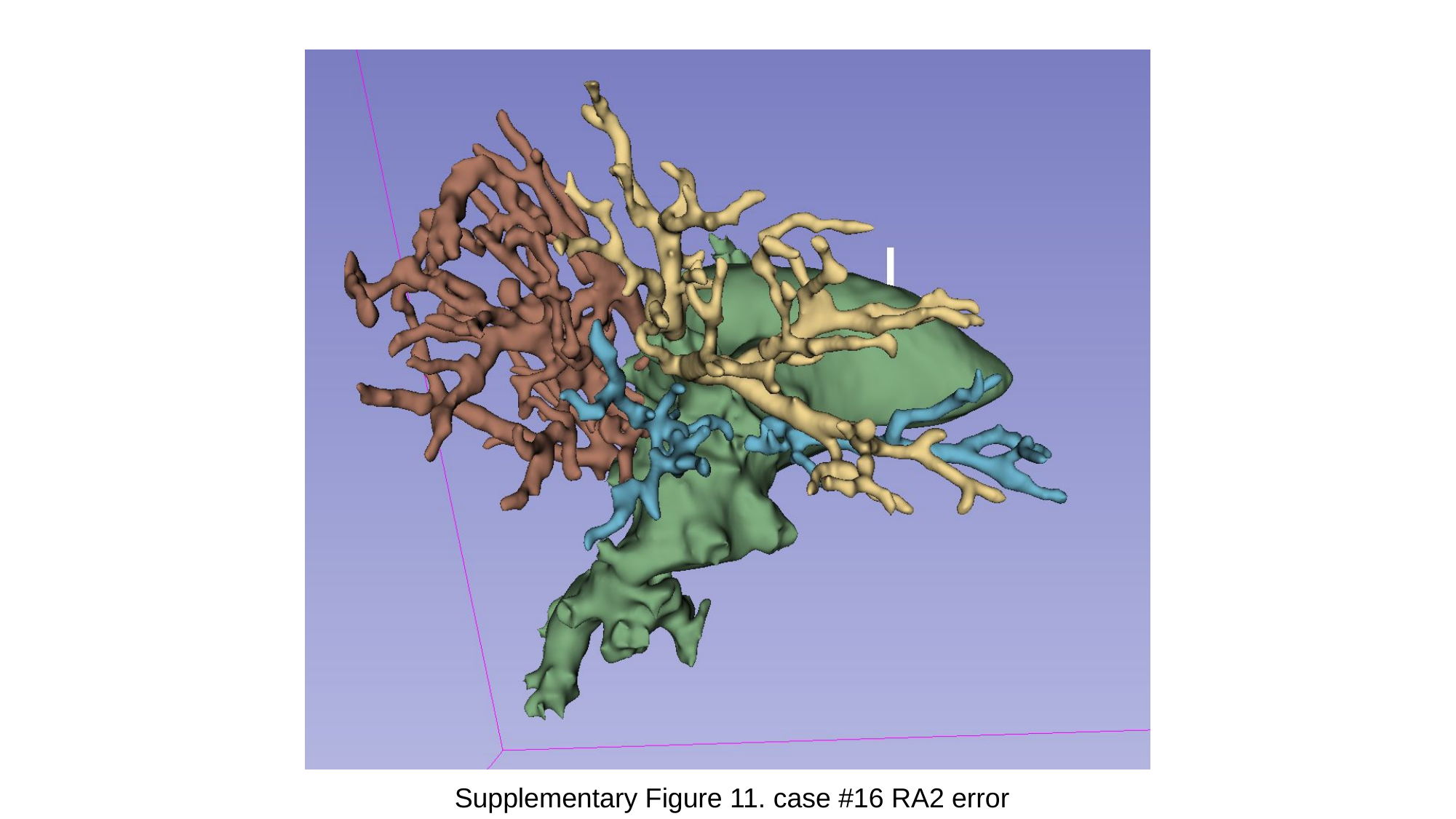

Supplementary Figure 11. case #16 RA2 error

## Slide 12
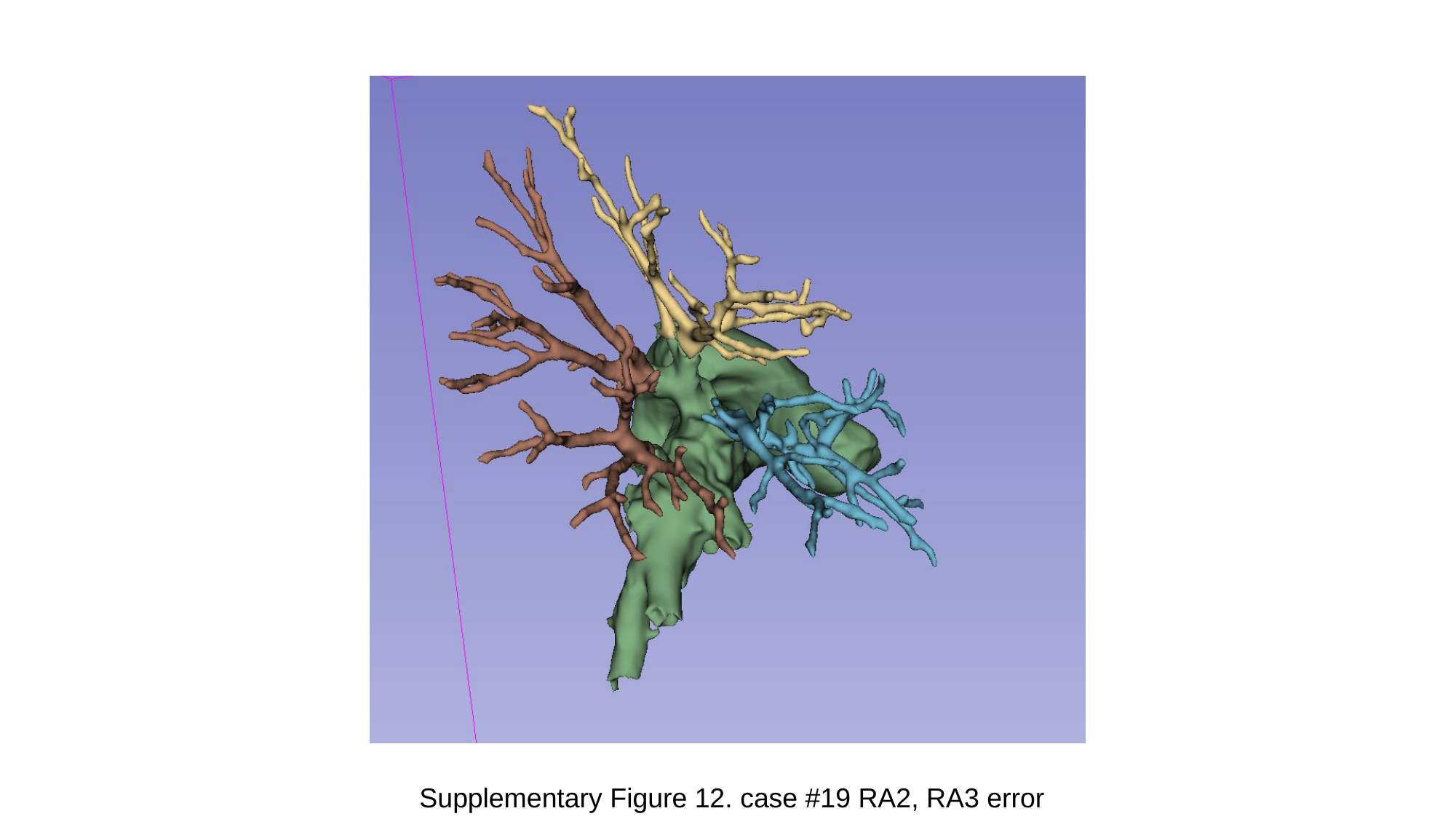

Supplementary Figure 12. case #19 RA2, RA3 error

## Slide 13
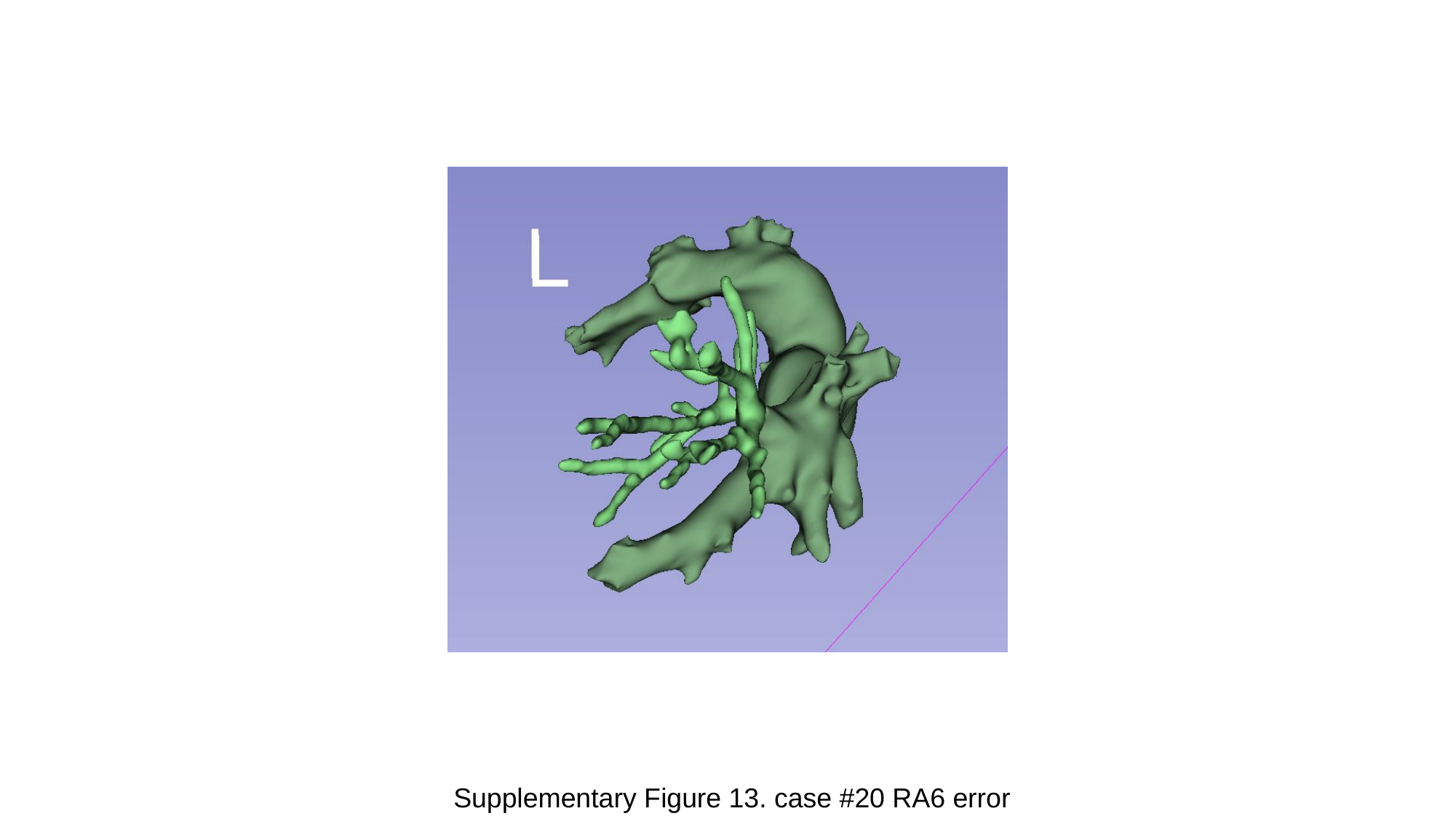

Supplementary Figure 13. case #20 RA6 error

## Slide 14
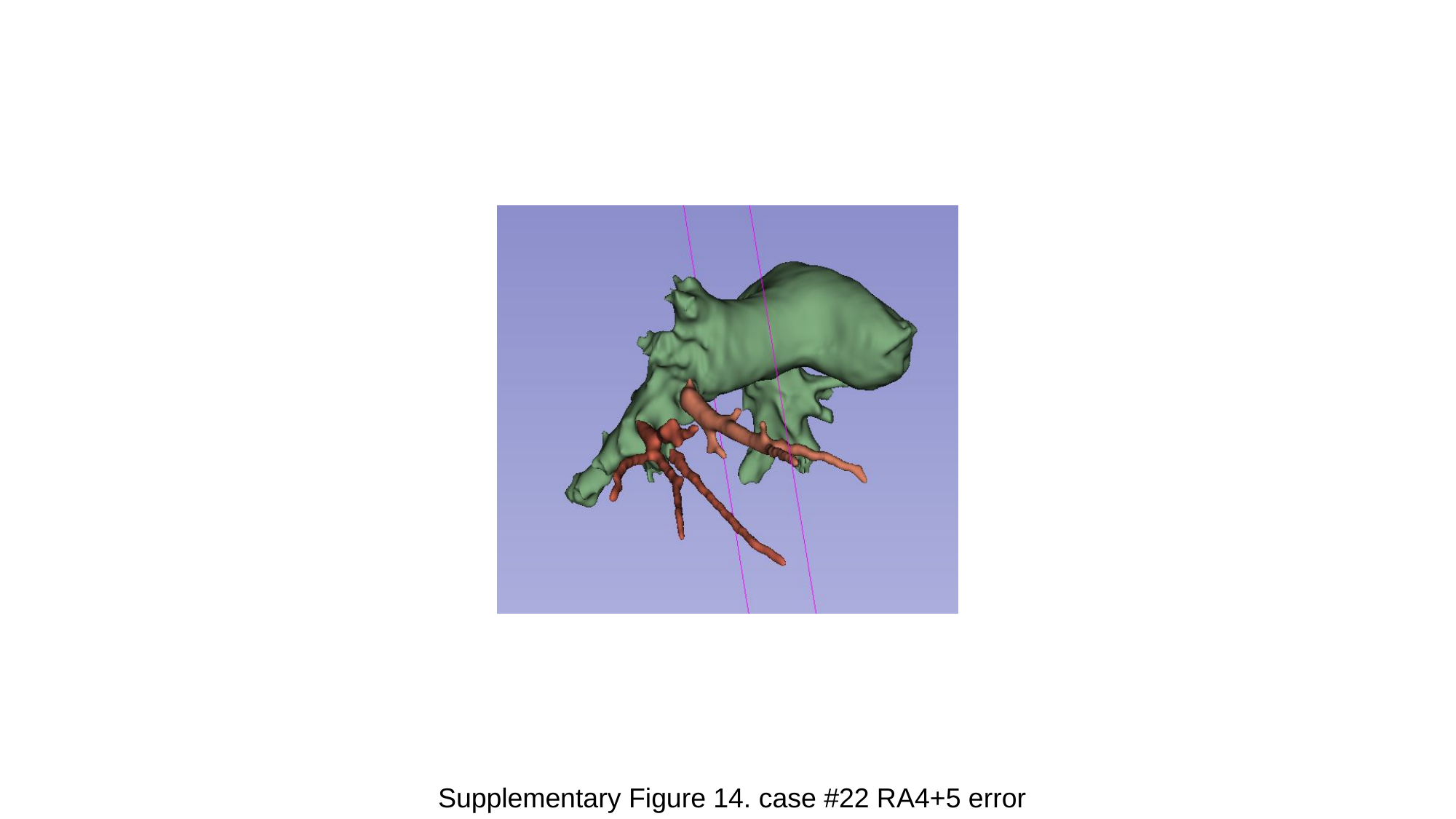

Supplementary Figure 14. case #22 RA4+5 error

## Slide 15
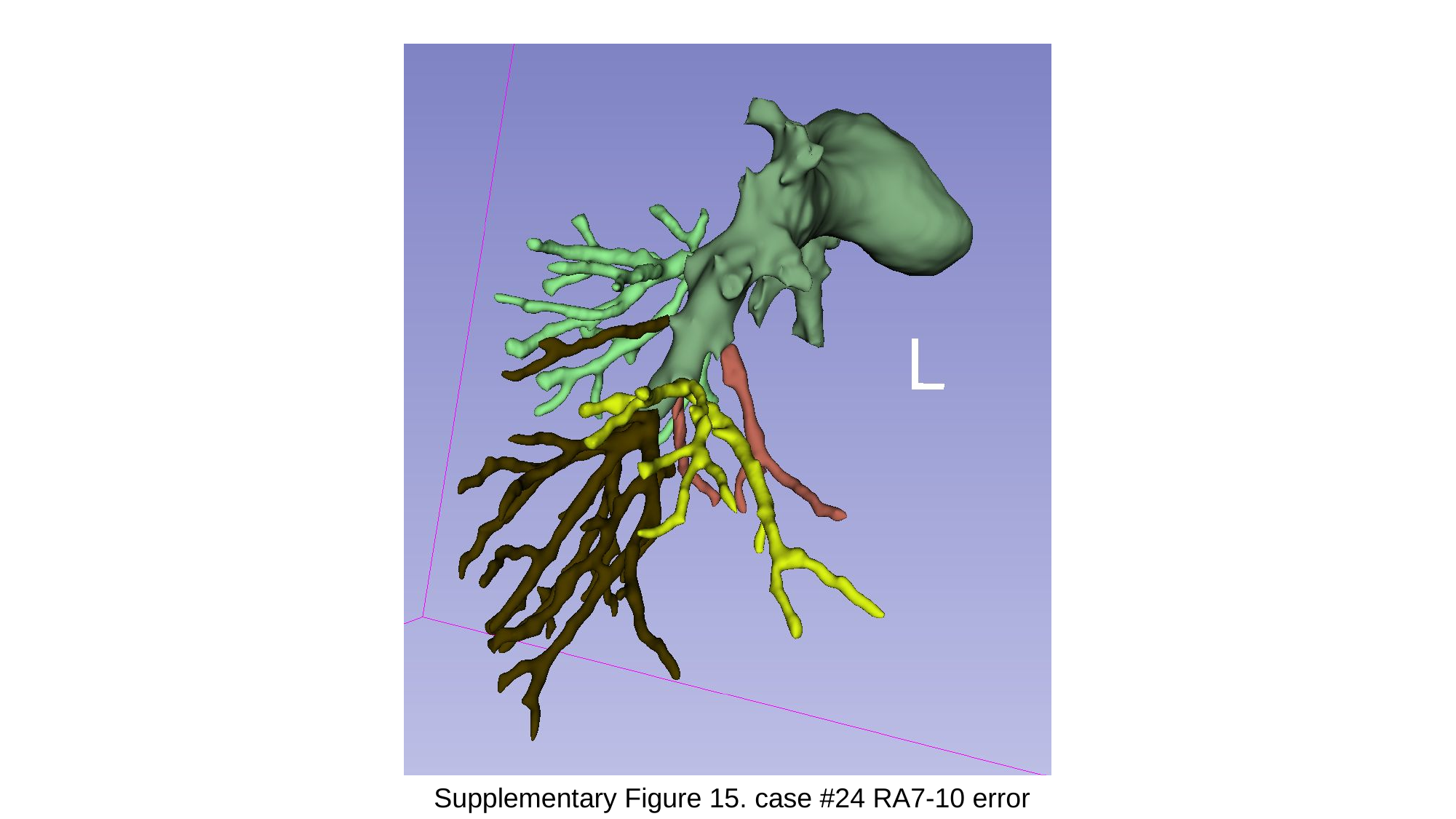

Supplementary Figure 15. case #24 RA7-10 error

## Slide 16
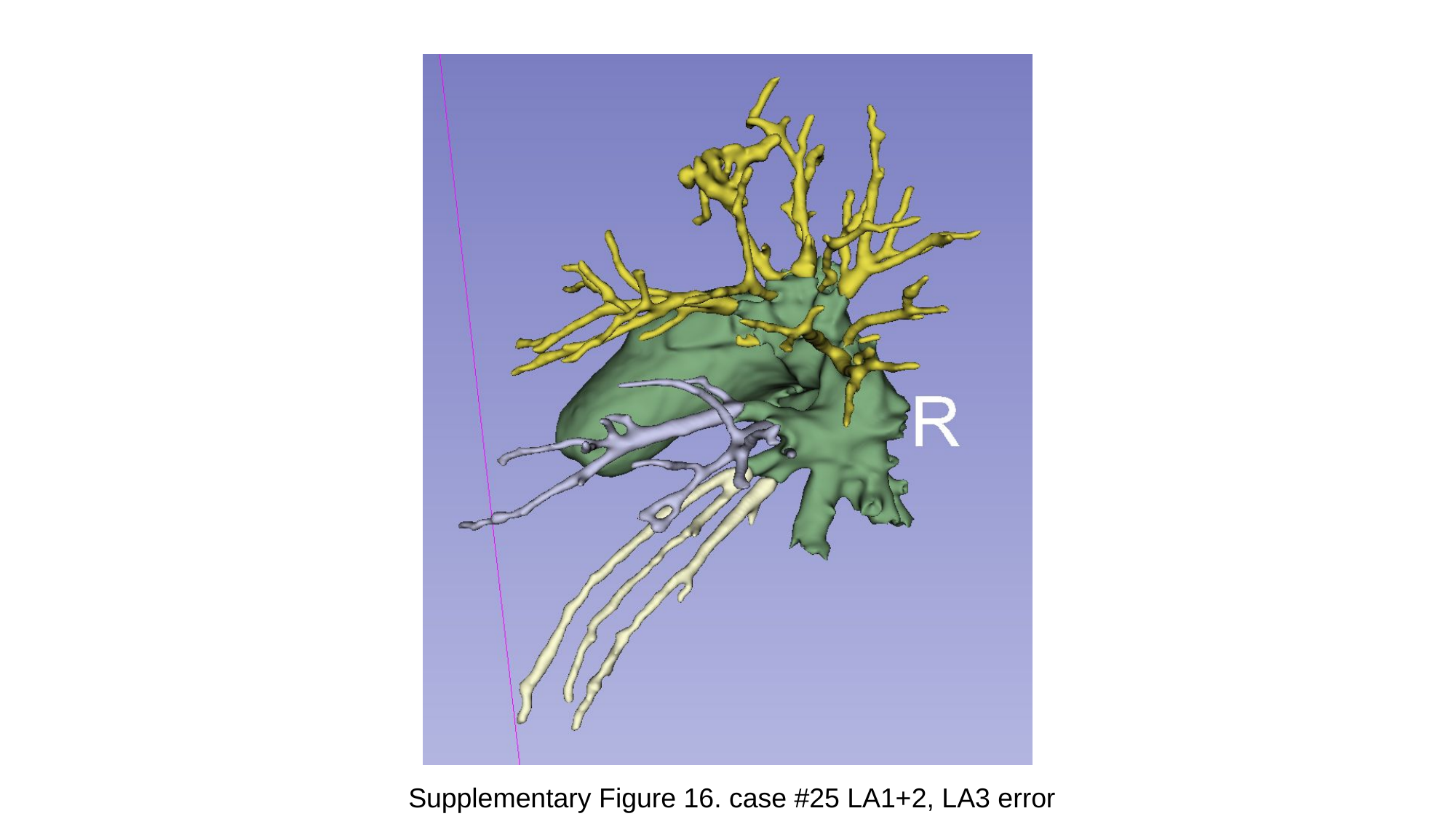

Supplementary Figure 16. case #25 LA1+2, LA3 error

## Slide 17
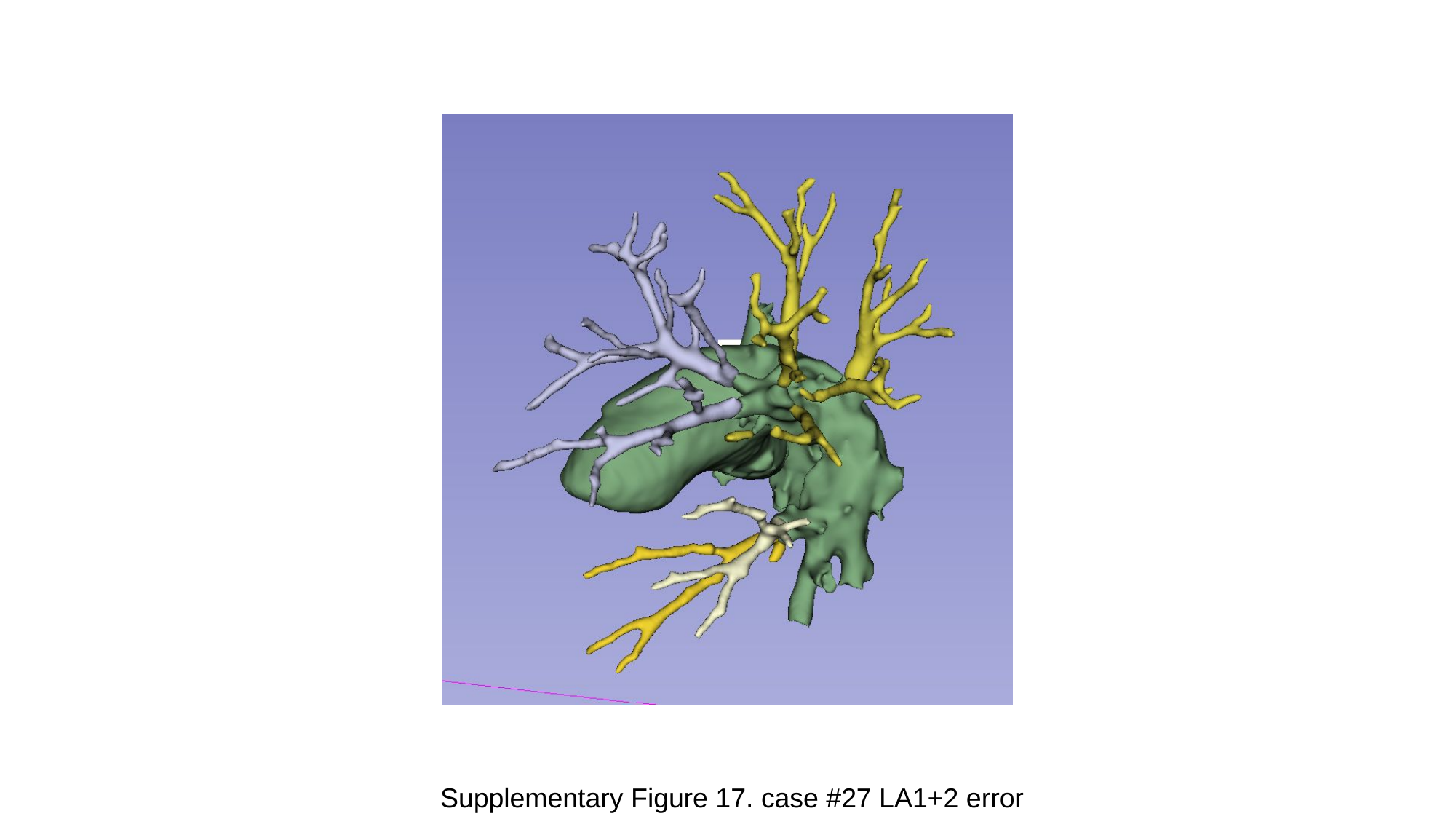

Supplementary Figure 17. case #27 LA1+2 error

## Slide 18
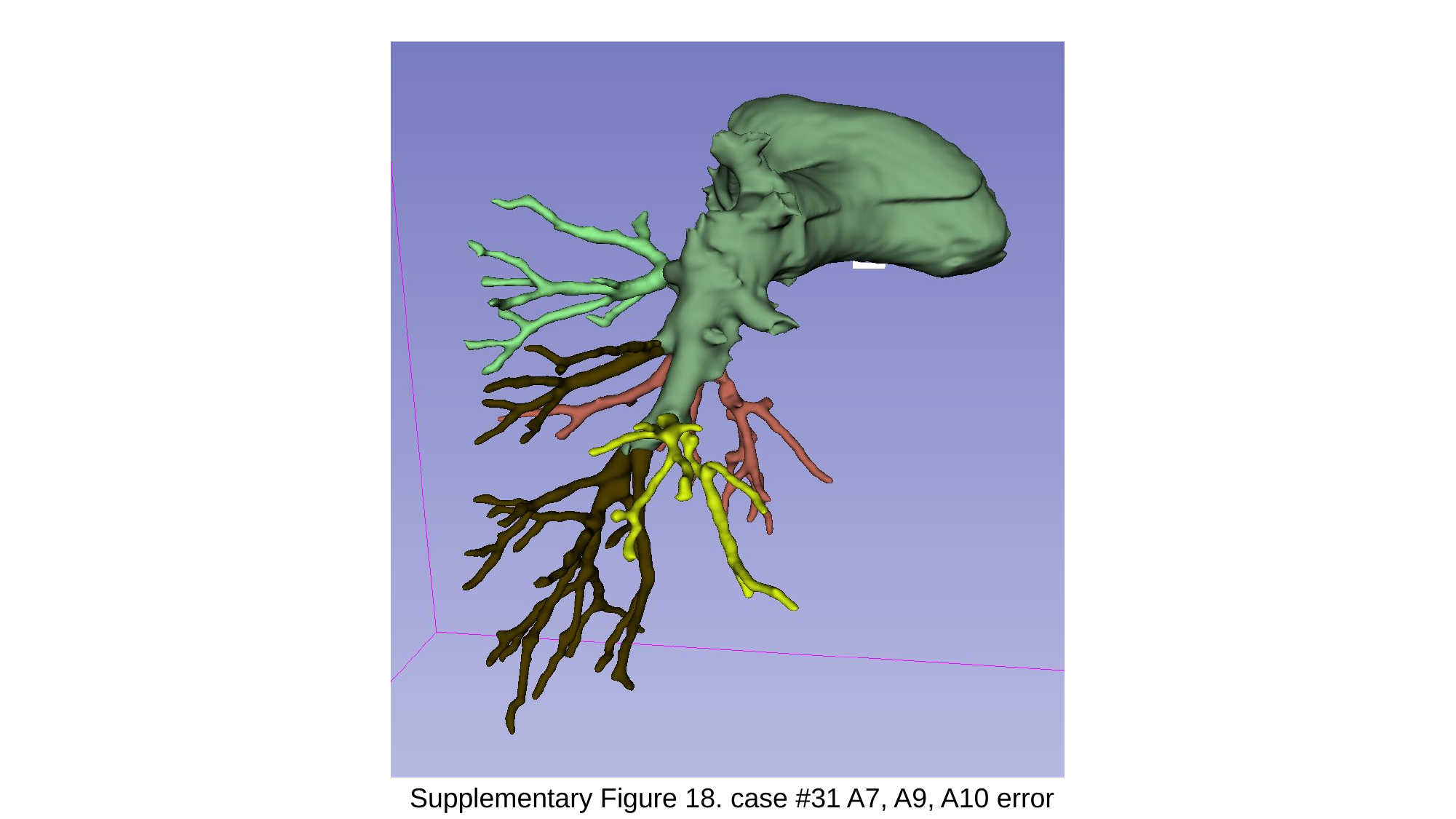

Supplementary Figure 18. case #31 A7, A9, A10 error
